# Modulation of the mechanism of action of antibacterial silver N-heterocyclic carbene complexes by variation of the halide ligand†

**DOI:** 10.1039/d4ra08093a

**Published:** 2025-01-20

**Authors:** Igor V. Esarev, Ingo Ott

**Affiliations:** a Institute of Medicinal and Pharmaceutical Chemistry, Technische Universität Braunschweig Beethovenstr. 55 38106 Braunschweig Germany ingo.ott@tu-bs.de

## Abstract

The antimicrobial properties of silver and silver complexes have been known in medicine since ancient times. However, limitations in stability and solubility have impaired medicinal chemistry and drug development research. With the advent of N-heterocyclic carbenes (NHC) as ligands, the development of synthesis methods for organometallic silver species of the type (NHC)AgX (where X = halide) has brought significant improvements, and the class of antimicrobial silver NHC complexes has emerged. However, reports studying structure–activity relationships and the mechanism of action of this compound type are still rare. This paper explores the activity of silver NHC complexes with halide (chloride, iodide) ligands and attempts to elucidate their mechanism of antibacterial action in Gram-negative *E. coli* bacteria in comparison to non-organometallic silver nitrate. In particular, the complexes with an iodide ligand were confirmed to cause stronger antibacterial effects in *E. coli* than silver nitrate. Moreover, iodide complexes exhibit an enhanced cellular uptake, show signs of DNA condensation, strongly inhibit TrxR in *E. coli* and cause a strong depolarization of the membrane potential and permeability of the inner cell membrane. In contrast, chloride silver NHC complexes and silver nitrate caused permeability of the outer membranes and also showed a different activity pattern in most of the studied mechanisms. In conclusion, by variation of the halide ligand of silver NHC complexes the mechanism of action and strength of antibacterial activity can be fine-tuned.

## Abstract 2

The halide ligand of silver N-heterocyclic carbene complexes governs their antibacterial activity and mechanism of action.

## Graphical abstract

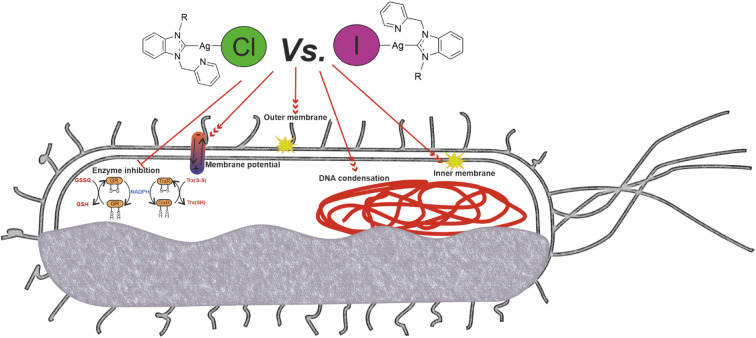

## Introduction

The antimicrobial properties of silver and its complexes have been known and used in medicine since ancient times.^1–3^ Whereas the antibacterial properties of this transition metal are nowadays used in many consumer products and medical devices, only a few silver-based drugs have reached the drug market. For example, silver nitrate and the silver complex of the sulfonamide antibiotic sulfadiazine are currently applied as topical antibiotics. The challenging physicochemical properties of many silver complexes, such as limited stability and low solubility, have certainly hampered the progress of silver complexes from basic research to advanced stages of drug discovery and development.

The formation of organometallic silver species by coordination of the metal to N-heterocyclic carbenes (NHCs) provided the opportunity to conveniently synthesize large numbers of more stable antibacterial silver compounds with great potential for metallodrug development and the literature on such compounds has been strongly increasing over the last few years.^4–11^

We have recently reported on antibacterial silver NHC complexes with halide ligands of the general type (NHC)AgX (X = Cl, Br or I) that showed potent inhibition of purified bacterial thioredoxin reductase (TrxR) and glutathione reductase (GR).^12^ Importantly, these complexes can be administered in biological medium with abundant chloride levels, which commonly diminish the biological activity of silver compounds as a consequence of the precipitation of insoluble silver chloride. The antibacterial effects of the complexes in general depended on the dynamic solution chemistry of the complexes and indicated a preference for complexes with iodide ligands due to their improved stability in solution, which was characterized by the higher prevalence of the (NHC)AgI monocarbene form over the [(NHC)_2_Ag]^+^[AgX_2_]^−^ biscarbene form with two NHC ligands. In addition, the derivatives with iodine as ligand showed a reduced toxicity towards eukaryotic cells that is highly desirable regarding the development of safe antibiotic drug candidates. The complexes could therefore serve as lead compounds for future organometallic silver drug candidates, however, their mechanism of action beyond the strong inhibition of the purified enzymes remained elusive.

The antibacterial mechanism of action of silver in metallic and ionic form is generally largely unknown, and the biological effects are commonly attributed to the release of free Ag^+^ ions from the drugs, nanoparticles or metallic surfaces. Main targets reported in the literature include the DNA, bacterial membranes or thiol-containing biomolecules, peptides and enzymes.^2,6,13–21^ Overall, the efficient antibacterial activity of silver species is most likely the result of a multimodal mechanism of action and such hypothesis has recently been strongly supported by proteomic studies.^22^

**Fig. 1:**
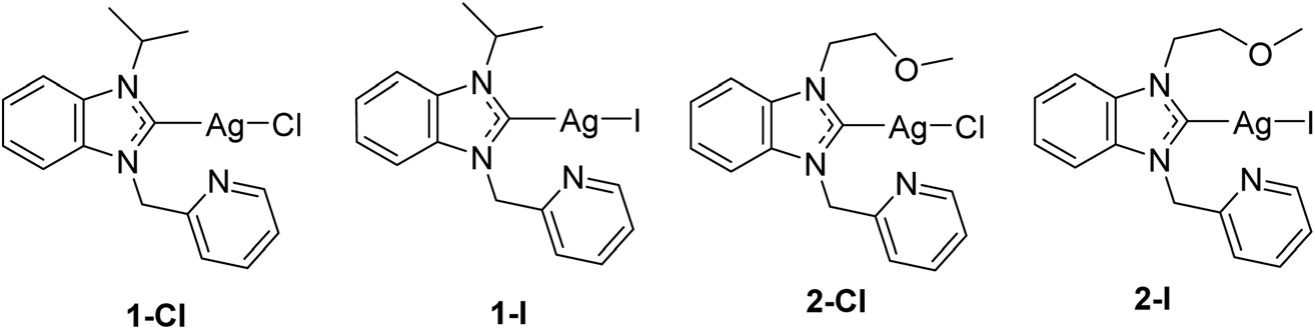
Structures of the investigated silver NHC halido complexes.^12^

In continuation of our previous report, which had confirmed different physicochemical properties in relation to the type of halide ligand, we selected 4 silver NHC halide complexes bearing chloride (1-Cl, 2-Cl) or iodide (1-I, 2-I) ligands for studies on their mechanism of action (see Fig. 1). The selected complexes contain two different NHC ligands, for which we had observed satisfactory solubility in our previous study with only minor differences in bioactivity in relation to the NHC ligand. With the type of NHC ligand not influencing the biological activity strongly, the chosen complexes provide a suitable selection to further evaluate the effects of the type of halide ligand on the mechanism of action. The studies were performed in the selected Gram-negative *Escherichia coli* strain DSM 1116.

## Results and discussion

### Antibacterial activity and uptake of silver into *E. coli* bacteria

**Fig. 2:**
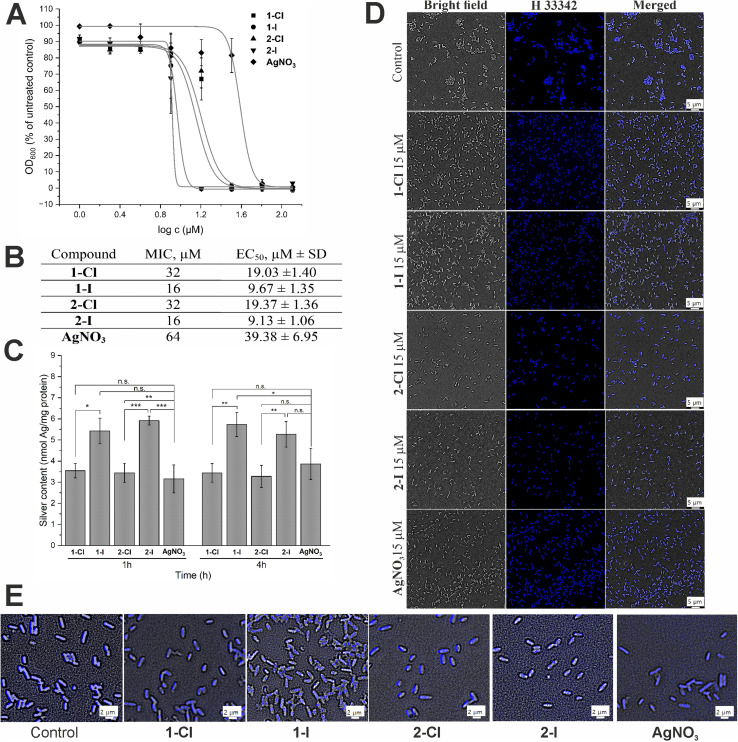
Antibacterial effects and cellular uptake of silver NHC complexes in comparison to silver nitrate. (A) Antimicrobial activity against *E. coli* DSM 1116 (*n* = 3). (B) Minimal inhibitory concentration (MIC, in μM) and EC_50_ (in μM ± standard deviation) values for *E. coli* DSM 1116 (*n* = 3). (C) Silver uptake in *E. coli* DSM 116 treated with 10 μM of silver complexes for 1 h and 4 h (*n* = 3). The symbols *, ** and *** indicate a significant difference between chloride and iodide analogues compared with each other and with AgNO_3_ (* – *p* < 0.05 and *** – *p* < 0.001, based on 95% Tukey's HSD test); n.s.: no significant difference observed (*p* > 0.05). (D) DNA staining in *E. coli* cells treated with 15 μM of the silver complexes for 1 h using Hoechst 33 342 probe. (E) Magnification of DNA stained *E. coli* for evaluation of DNA condensation.

We have recently determined the antibacterial activity of the selected complexes 1-Cl/I and 2-Cl/I in a panel of Gram-positive and Gram-negative bacteria.^12^ In this report, we focused on the Gram-negative *E. coli* strain and re-evaluated the activity of freshly synthesized complexes under similar experimental conditions and obtained comparable results (Fig. 2A and B), confirming a good reproducibility of the previous results. Generally, for the iodide silver NHC complexes 1-I and 2-I the EC_50_ values were approximately half of those of the respective chloride analogues 1-Cl and 2-Cl, and all silver NHC complexes were more active than the reference silver nitrate.

Since the antibacterial effect of silver compounds is attributed to the amount and the release rate of Ag^+^ ions, knowledge on the accumulation of silver in the bacteria is important for better understanding of differences in the biological activity of individual silver compounds.^23^ Here we determined the silver levels of *E. coli* cells treated with 10 μM of the silver NHC complexes or silver nitrate by a method based on high resolution continuum source atomic absorption spectroscopy (see Fig. 2C). Generally, the uptake of silver occurred fast and the bacteria contained high levels of the metal after only 1 h of exposure, which did not significantly differ from the values obtained after 4 h. Interestingly, the amount of silver delivered by the iodide analogues 1-I and 2-I was significantly higher than the accumulation of the metal after treatment with silver nitrate or chloride silver NHC complexes 1-Cl and 2-Cl. Certainly, this could contribute to explaining the higher activity of the silver NHC complexes with the iodide ligand. However, the silver levels obtained with the chloride silver NHC complexes 1-Cl and 2-Cl were similar to those for silver nitrate, which had the lowest antibacterial activity of all studied silver species (see Fig. 2B). Hence, the amount of silver accumulated in the bacteria alone could not explain the differences in the biological activity.

To evaluate the effect of silver complexes on DNA functionality, we used the fluorescent minor-groove binder dye Hoechst 33 342. The silver complexes were applied at a dosage of 15 μM and compared with an untreated control (Fig. 2D). In general, all silver treated bacteria showed strong blue fluorescence that was, however, less strong than that the emission of the untreated control indicating some interference with Hoechst binding to the DNA. In particular, *E. coli* cells treated with the iodide complexes 1-I and 2-I showed signs of DNA condensation (Fig. 2E), which is indicative of a leakage of cytoplasmic contents after cell lysis due to increased cell membrane permeability.^24,25^ Taken together, binding to the DNA and DNA damage by the silver compounds can be considered to contribute to the antibacterial effects of the silver complexes.

### Intracellular thioredoxin reductase (TrxR) inhibition and glutathione (GSH) depletion

**Fig. 3:**
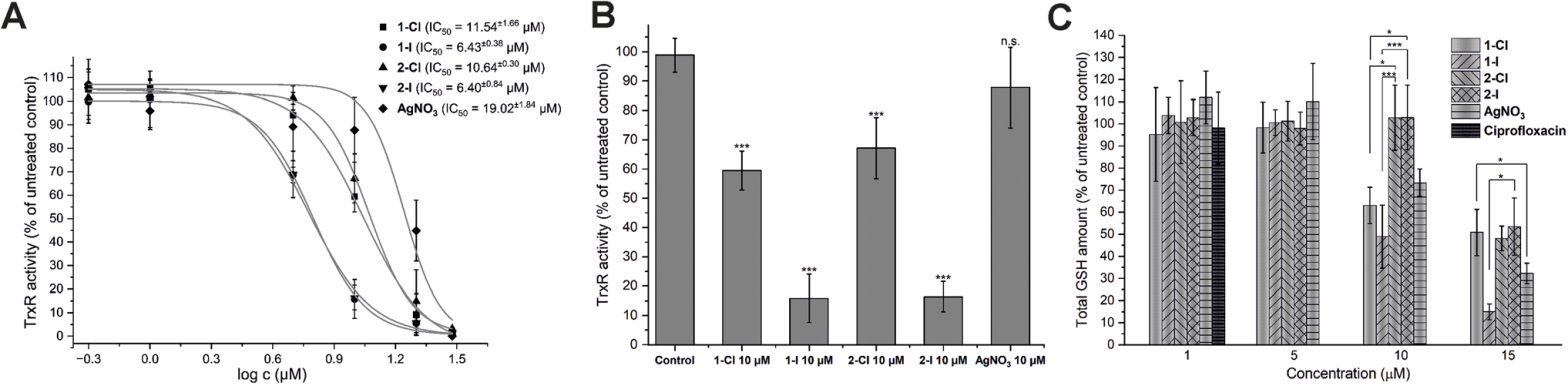
Silver complexes disrupt activity of TrxR and GSH activity in *E. coli* DSM 1116. (A) Concentration-dependent inhibition of TrxR in DTNB reduction kinetic assay with IC_50_ for each compound (*n* = 3). (B) Inhibition of TrxR by 10 μM of silver compounds (*n* = 3). (C) Depletion of glutathione levels in *E. coli* cells after treatment with silver complexes at different concentrations. Ciprofloxacin at 1.0 μM was used as negative control (*n* = 3). The symbols * and *** indicate a significant difference between cell samples treated with different compounds (*p* < 0.05 and *p* < 0.001 respectively, based on 95% Tukey's HSD test). For the other comparisons, no significant difference was observed (n.s., *p* > 0.05).

As previously reported, silver NHC complexes 1-Cl/I and silver nitrate are potent inhibitors of purified disulfide reductases (*i.e.* glutathione reductase, thioredoxin reductase) with IC_50_ values in a narrow submicromolar concentration range (0.03–0.12 μM).^12^ Taken together with the above-described high silver levels in *E. coli* exposed to the silver compounds, it was therefore of interest to evaluate if the high enzyme inhibitory activity would translate into enzyme inhibition in living bacteria. Accordingly, the effects of the silver complexes on TrxR activity in *E. coli* were determined (Fig. 3A and B). At a concentration of 10 μM of the silver complexes, the iodide derivatives 1-I and 2-I strongly inhibited TrxR activity in *E. coli* to less than 20% compared with the untreated control, while the chloride analogues 1-Cl and 2-Cl were much less active (TrxR activity 60–70%), and silver nitrate did not significantly affect TrxR activity (approx. 90% remaining activity). The higher activity of the complexes with iodide ligands compared with the chloride analogues was also reflected in the respective IC_50_ values of the complexes (approx. 6 μM for 1-I/2-I*vs.* approx. 11 μM for 1-Cl/2-ClFig. 3A) and was in general in excellent agreement with the results from the antibacterial activity and cellular uptake studies.

The inhibition of thioredoxin reductase in bacteria lacking glutathione has been considered as a mechanism of antibiotic activity.^26^ Since the silver compounds of this report have been confirmed to be dual inhibitors of both TrxR and glutathione reductase (GR), it was of interest to study if a possible glutathione depletion could provide a synergistic mechanism of action in *E. coli*.^12^ Accordingly, the bacterial glutathione levels of *E. coli* incubated with the silver compounds in concentrations from 1.0 to 15 μM were determined (Fig. 3C). While none of the silver compounds was active at 1.0 and 5.0 μM, at 10 μM complexes 1-Cl and 1-I with a lipophilic isopropyl side chain were able to trigger a significant decrease of glutathione levels, in contrast to the more hydrophilic analogues 2-Cl and 2-I with the methyloxyethyl side chain. Interestingly, silver nitrate has also caused a decrease of the glutathione level at the same concentration. At 15 μM, all silver compounds led to a significant GSH depletion with 1-I being the most active example. Notably, the effects of the silver NHC complexes on the glutathione levels were not strongly governed by the nature of the halide counterions as observed with TrxR inhibition in the bacteria. However, GSH depletion in *E. coli* by the silver NHC complexes was evident at comparable concentration range as TrxR inhibition, supporting the hypothesis that they act as dual inhibitors of TrxR and GR in *E. coli*. On the contrary, silver nitrate triggered its effects primarily *via* GSH depletion.

### Effects of silver compounds on membranes in *E. coli* cells

Bacterial membranes have been investigated as possible targets of the antibacterial action of silver species.^14,21^ To evaluate possible pathways of membrane damage caused by silver NHC complexes, various assays widely used for the evaluation of effects on membrane potential^27^ as well as damage of outer^28^ or inner^29^ membranes were applied.

**Fig. 4:**
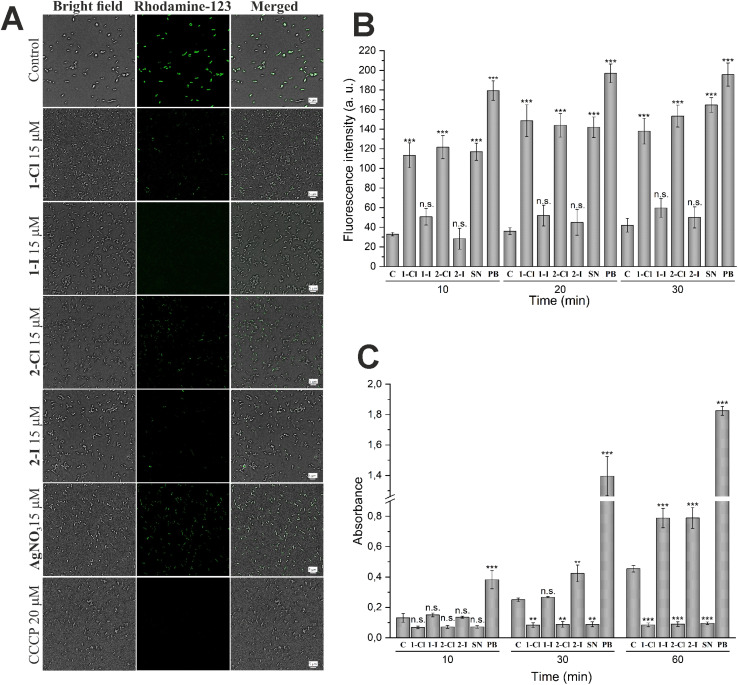
Differences in effects of silver containing compounds on cellular membranes in *E. coli.* (A) The depolarization of membranes in *E. coli* cells was determined using the rhodamine 123 probe. Cells were treated with 15 μM of selected compound for 1 h. CCCP at 20 μM was used as positive control. (B) Evaluation of outer membrane damage by NPN uptake in *E. coli* DSM 1116 by silver NHC complexes and silver nitrate (SN) at 30 μM and polymyxin B at 10 μM (PB, positive control) at different time points (*n* = 3). (C) Evaluation of inner membrane permeabilization by ONPG in *E. coli* DSM 1116. Cells were incubated with ONPG in the presence of silver NHC complexes and silver nitrate (SN) at 30 μM or polymyxin B at 10 μM (PB, positive control) at different time points (*n* = 3). Each sample is compared with untreated control (C) by 95% Tukey's HSD test (n.s. = not significant, * = *p* < 0.05, ** = *p* < 0.01, *** = *p* < 0.001).

First, the disturbance of membrane potential was examined using rhodamine 123 staining.^26^ The obtained fluorescent images (see Fig. 4A) revealed a significant depolarization of *E. coli* membrane potentials after treatment with the silver complexes, as evidenced by the decrease of fluorescence intensity. Interestingly, this effect was strongest for the silver iodide NHC complexes 1-I and 2-I and supports their higher antibacterial activity in comparison to other silver compounds used in this report.

For the evaluation of outer membrane permeability, the assay based on the uptake of the fluorescent probe 1-*N*-phenylnaphthylamine (NPN) was performed.^27^ Partitioning of NPN into the hydrophobic interior of the outer membrane causes an increase of fluorescence emission. The results shown in Fig. 4B indicate a significantly enhanced permeability of the outer membrane in cells treated with silver chloride NHC complexes 1-Cl and 2-Cl as well as silver nitrate. In contrast, the silver iodide derivatives 1-I and 2-I did not trigger a significant effect.

Together with the outer membrane damage assay, we also have carried out the inner membrane damage assay. The hydrolysis of the weakly permeable *o*-nitrophenyl-β-galactoside (ONPG) by β-galactosidase in the cell cytoplasm is indicative of an enhanced permeability of the inner cell membrane.^28^ As shown in Fig. 4C, both silver iodide NHC complexes 1-I and 2-I showed a significant time-dependent increase of the signal confirming the strong activity of the two compounds in this assay. In contrast, the chloride analogues 1-Cl and 2-Cl and silver nitrate over time caused a significant decrease of the signal intensity compared with the untreated control, indicating that the complexes might be inhibitors of the OPNG hydrolyzing β-galactosidase and thus could not be evaluated by the used method.

In fact, in a report of Martino *et al.*, silver ions had been determined as competitive inhibitors of β-galactosidase from mammalian cells.^30^ We tested whether the observed signal depletion could be caused by interaction with intracellular bacterial β-galactosidase using lysed cells (Fig. S1†). As anticipated, 1-Cl, 2-Cl and silver nitrate completely blocked OPNG hydrolysis, while the iodide analogues 1-I and 2-I were much less active.

## Conclusions

**Fig. 5:**
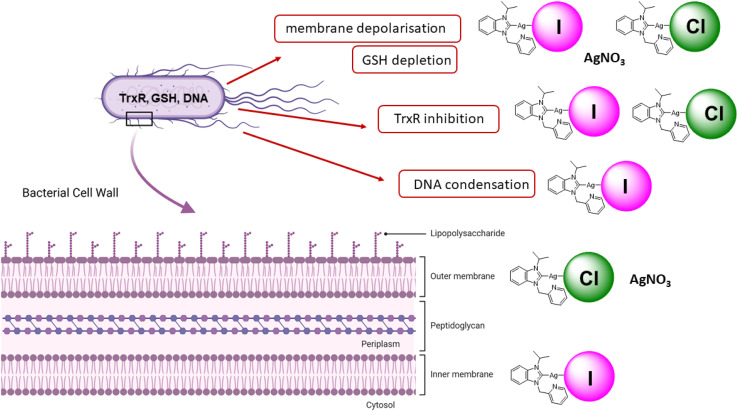
Overview on the mechanisms of action of iodide and silver NHC complexes.

While silver NHC complexes have been widely evaluated as antibacterial drug candidates, studies on their mechanism of action as well as structure–activity relationships are rare. In this report, silver NHC complexes with halide (chloride, iodide) ligands were confirmed to cause stronger antibacterial effects in *E. coli* than silver nitrate with the iodide analogues having the strongest activity. The enhanced activity of the iodide silver NHC complexes in comparison to their chloride analogues correlated with an increased uptake of silver into the bacteria, signs of DNA condensation, increased inhibition of TrxR in *E. coli*, a stronger depolarization of the membrane and permeability of the inner cell membrane. The favorable properties of the iodide complexes could be related to the distinct coordinative properties of iodide as larger, less electronegative, better polarizable and more Lewis-soft ligand in comparison to chloride. Higher lipophilicity of the iodide complexes compared with the chloride complexes would also explain their higher silver accumulation in the bacteria. In relation to the inhibition of TrxR, both chloride and iodide silver NHC complexes caused a strong depletion of GSH indicating that they act as dual inhibitors of TrxR and glutathione reductase systems. With such property, they differ from silver nitrate, which showed comparable GSH depletion yet with the lower activity as an inhibitor of TrxR in *E. coli*. The chloride silver NHC complexes and silver nitrate caused permeability of the outer membranes. In contrast, the iodide analogues were inactive in this aspect. Fig. 5 shows an overview of the antibacterial properties of the silver NHC in a simplified comparative manner. Further experiments on OPNG hydrolysis indicated that in particular silver nitrate and the silver NHC complexes could be inhibitors of bacterial β-galactosidase. Taken together, this report provides a rare example of the significant influence of small structural changes on organometallic silver complexes and provides valuable insights into the modulation and fine-tuning of their properties as antibacterial drug candidates by variation of the coordinated halide ligand.

## Materials and methods

### General

All reagents and materials were purchased from Merck, TCI or BLDpharm and used without additional purification steps. Absorption measurements were performed on a PerkinElmer 2030 Multilabel Reader VICTOR™ X4. Fluorescence microscopy was performed using a Keyence BZ-X810 fluorescence microscope equipped with an 100× apochromatic oil-immersion objective lens. The synthesis of silver complexes used in this study was previously described.^12^ All compounds are >95% pure by elemental analysis. All solutions were prepared using ultrapure water (Arium® Pro Ultrapure Water Systems, Sartorius).

### Culture of bacteria

Gram-negative strain *Escherichia coli* (DSM1116, ATCC 9637) was obtained from DSMZ (German Collection of Microorganisms and Cell Cultures GmbH, Braunschweig, Germany) as a lyophilized cell pellet. The pellet was resuspended in Müller Hinton broth (MHB, 21 g L^−1^, pH 7.4) containing 10% glycerol, aliquoted into cryovials, and they were kept frozen over liquid nitrogen. This frozen stock was then used to deliver bacterial cells on a Müller–Hinton agar plate (MHA, 38 g L^−1^, pH 7.0) by use of an inoculation loop, and the plate was incubated at 37 °C for 24 h to obtain bacterial colonies.

### Antibacterial activity

The bacterial suspension of *E. coli* DSM 1116 was prepared by overnight incubation of a single colony at 37 °C in MHB. Minimum inhibitory concentration (MIC) and EC_50_ values were determined following a standardized protocol in broth microdilution assays.^31^ In short: the compounds were dissolved as stock solutions in DMSO and diluted with MHB to concentrations in the range of 128 to 2.0 μM (final DMSO concentration: 1%, final OD_600_: 0.0005). The serial dilutions of test compounds were prepared in duplicate. After incubation of the plates for 22 h at 37 °C, the absorbance at 600 nm was measured using a PerkinElmer 2030 Multilabel Reader VICTOR™ X4 microplate reader. MIC (the lowest concentration of compound that completely inhibits the cell growth) and EC_50_ (the concentration, at which 50% of effect of compound on cell growth is observed) of the tested compounds were determined in three independent experiments by dose–response curve fitting with Origin Pro (OriginLab Corp.).

### Cellular uptake studies in *E. coli* cells

For cellular accumulation studies, the overnight culture of *E. coli* DSM 1116 was diluted and grown to mid log phase (OD_600_ = 0.5). The 10 ml of cell suspensions were treated with selected test compounds (final DMSO concentration: 0.1%; final test compound concentration: 10 μM). After 1 h and 4 h of exposure, cells were isolated by centrifugation at 3500*g* at r.t. for 10 min. The cells were washed three times with 8 ml of PBS buffer (pH 7.4). The obtained cell pellets were stored at −20 °C for further use. All experiments were carried out in triplicate.

### Metal quantification in cell lysates by HRCS-AAS

Dry cell pellets were diluted with 200 μl of lysis buffer (50 mM Tris·HCl, 2 mM EDTA, 100 mM NaCl, 20 mM sodium β-glycerophosphate, 0.1% Triton X-100) and lysed for 2 hours in an ultrasonic bath (240 W, 65 Hz). The protein content was determined *via* Bradford assay. The silver quantification was performed using a high-resolution continuum source atomic absorption spectrometer ContrAA® 700 (Analytik Jena AG). A pure sample of AgNO_3_ was used as standard, and calibration was done in a matrix-matched manner (meaning all samples and standards were adjusted to a protein concentration of 1 mg ml^−1^ by dilution with ultrapure water). The calibration and samples were measured in duplicate. The final cellular silver concentrations were calculated from data obtained in three independent experiments and are expressed as nmol of metal per mg of cell protein. Sample preparation: Triton-X 100 (1%, 10 μl), as well as nitric acid (13%, 10 μl), were added to each calibration standard and probe (100 μl). A volume of 15 μl was injected into a coated standard graphite tube (Analytik Jena AG) and thermally processed as described in Table S1.† Silver was determined at a wavelength of 328.1 nm.

### Fluorescence microscopy staining

Overnight grown *E. coli* bacteria were diluted and grown until an OD_600_ of at least 0.4 was reached. A volume of 2 ml of bacterial suspension was incubated with 2 μl of 15 mM stock solutions of the test compounds or silver nitrate in DMSO (final concentration of compounds 15 μM, final DMSO concentration 0.1%) as well as respective positive controls for 1 h at 37 °C. The cells were then harvested by centrifugation (4 °C, 3500*g*, 5 min.) and washed 2 times with 1 ml of ice-cold phosphate buffered saline (PBS, pH 7.4). The resulting pellet was suspended in 2 ml PBS, and the respective fluorescent dye was added. For DNA imaging, 2 μl of 10 mM Hoechst 33 342 solution in DMSO was added, and cells were incubated for 30 min at 37 °C. Similarly, for the detection of the cell membrane potential, 2 μl of 10 mM rhodamine 123 solution in DMSO was added and the cells were incubated for 30 min at 37 °C. After incubation, cells were again harvested by centrifugation (4 °C, 3500*g*, 5 min) and washed once with PBS, 2 times with sterile ultrapure water, and were finally resuspended in 2 ml of sterile water. 10 μl of the cell suspension was then distributed onto glass slide. After drying, cells were covered with a cover slip, and the fluorescence was visualized using a Keyence BZ-X810 fluorescence microscope equipped with 100× Apochromat objective lens (DAPI channel for Hoechst 33 342 staining, *λ*_ex_ = 358 nm, *λ*_em_ = 461 nm; GFP channel for rhodamine 123, *λ*_ex_ = 395 nm, *λ*_em_ = 509 nm) (Keyence, Japan). All experiments were repeated three times independently.

### Preparation of cell lysates for enzyme activity measurements and determination of the protein content

The bacterial suspension of *E. coli* DSM 1116 was prepared by overnight incubation of a single colony at 37 °C in MHB. The suspension was diluted and further grown in MHB to mid log phase (OD_600_ = 0.4). 2 ml of cell suspensions were incubated together with 2 μl of DMSO solutions of the test compounds at several selected concentrations for 1 h at 37 °C (final DMSO concentration 0.1%). The cells were then collected by centrifugation at 3500*g*, 4 °C for 5 min. The supernatant was discarded, the dry cell pellets were washed 3 times with ice-cold PBS. The cells were further resuspended in 200 μl of cell lysis buffer (50 mM Tris·HCl, 2 mM EDTA, 100 mM NaCl, 20 mM sodium β-glycerophosphate, 0.1% Triton X-100), mixed with 2 μl 100× DMSO solution of EDTA-free protease inhibitor cocktail (Abcam, article ab270055) and lysed in an ultrasonic bath (240 Hz, 65 W) for 5 min on ice. The resulting suspension was centrifuged at 21 500*g*, 4 °C for 20 min, and the supernatant was transferred to a clean tube. For protein quantification, 20 μl of each sample was diluted 1 : 1 with ultrapure water, and 20 μl of diluted sample was added to 200 μl of Bradford reagent (25 mg Serva Blue G (Merck) in 25 ml ethanol 96%, 25 ml ultrapure water and 50 ml H_3_PO_4_ 86%, stored at −20 °C and freshly diluted 1 + 4 with ultrapure water prior to use), incubated in a 96-well plate while shaking at room temperature for 30 min, and read using a PerkinElmer Victor X4 plate reader at a wavelength of 595 nm. The measurements were performed in duplicate, and the mean was used to calculate the protein content. For a standard calibration curve, bovine serum albumin (Merck) was used in graded concentrations (0 to 1 mg ml^−1^ protein).

### Determination of TrxR activity in *E. coli* cell lysates

The activity of bacterial TrxR in cells was determined *via* DTNB-based assays as described previously.^26,32^ Aliquots of the lysate corresponding to 35 μg of cellular protein (approximately 35 μl of lysate) were mixed the with 10 μl of 1 mM NADPH solution in TE buffer (50 mM Tris·HCl, 1 mM EDTA, pH 7.6), 5 μl of thioredoxin from *E. coli* (Trx, Merck, 1191 μg mL^−1^) (for positive control and cell extracts treated with test compounds) or 5 μl of TE buffer (for negative control) in a well of 96 well-plate. The resulting solutions were incubated for 15 min at 25 °C. After the incubation, 25 μl of DTNB–NADPH mixture (6 mM of DTNB, 200 μM of NADPH in TE buffer) was added to reach 2 mM DTNB, 200 μM NADPH and 6.5 μM Trx in test solution. After thorough mixing, the absorbance at 405 nm due to formation of 5-TNB was monitored by PerkinElmer 2030 Multilabel Reader VICTOR™ X4 in 30 s intervals (10 measurements). The TrxR activity was corrected by subtraction of the negative control absorption values. The increase in concentration of 5-TNB followed a linear trend (*R*^2^ ≥ 0.990) and the enzymatic activities were calculated as the slope (increase in absorbance per second) thereof. The inhibition is presented as the mean IC_50_ values and standard deviations obtained in three independent experiments.

### Determination of total glutathione amount in *E. coli* cell lysates

The activity of total glutathione in cells was determined *via* DTNB-based assays as described previously.^26,32^ 35 μg of cellular protein (approximately 35 μl of lysate) was mixed the with 10 μl of 1 mM NADPH solution in TE buffer, 5 μl of glutathione reductase from *S. cerevisiae* (GR, Merck, stock concentration in 0.25 μM) (for positive control and cell extracts treated with test compounds) or 5 μl of TE buffer (for negative control) in a well of 96 well-plate. The resulting solutions were incubated for 20 min at 25 °C. After the incubation, 25 μl of DTNB–NADPH mixture (6 mM of DTNB, 200 μM of NADPH in TE buffer) was added to reach 2 mM DTNB, 200 μM NADPH and 0.025 μM of GR in test solution. After thorough mixing, the absorbance at 405 nm due to formation of 5-TNB was monitored by PerkinElmer 2030 Multilabel Reader VICTOR™ X4 in 30 s intervals (15 measurements). The absorbance was corrected by subtraction of the negative control absorption values. The GSH level is presented as activity in % of positive control with standard deviations calculated from three independent experiments.

### Outer membrane permeability assay

The damage of outer membrane was determined by well-established procedure measuring the uptake of *N*-phenylnaphthalen-1-amine (NPN).^28^ Briefly, the overnight culture of *E. coli* DSM 1116 was diluted and grown to mid log phase (OD_600_ = 0.5). A volume of 1.0 ml of the culture was transferred to Eppendorf tubes, and the suspension was centrifuged at 2500*g* at r.t. for 10 min. The resulting cell pellet was washed three times with 1.0 ml of 5 mM Hepes buffer containing 5 mM glucose (pH = 7.2). The cells were resuspended in 1 ml of the same buffer, and 100 μl of this suspension was mixed with 100 μl of buffer containing 20 μM NPN in a well of a 96-well plate to obtain a solution with OD_600_ 0.25 containing 10 μM NPN, which was further incubated for 30 min at r.t. To the resulting mixture (200 μl), 2.0 μl of DMSO solution free of compounds (negative control), 3 mM compounds stock solution in DMSO (test solution, 30 μM, final DMSO concentration is 1%) or 1 mM aqueous solution of polymyxin B sulfate (from Merck, positive control, 10 μM) was added. Fluorescence was measured using a PerkinElmer 2030 Multilabel Reader VICTOR™ X4 at an excitation wavelength of 355 nm and an emission wavelength of 435 nm for 30 min. The selected signals after 10, 20 and 30 min of the first measurement are presented as fluorescence intensity with a subtracted value of buffer with NPN. The values are given with standard deviations obtained from three independent experiments.

### Inner membrane permeability assay

The increase of inner membrane permeability caused by test compounds is determined by measuring the cytoplasm β-galactosidase activity *via* hydrolysis of *o*-nitrophenyl-*b*-d-galactopyranoside (ONPG) according to the previously published procedure with minor modifications.^33^ Briefly, the overnight culture of *E. coli* DSM 1116 grown in MHB containing 2% lactose was diluted in the same medium and grown to mid log phase (OD_600_ = 0.5). A volume of 1.0 ml of the culture was transferred to Eppendorf tubes, and the suspension was centrifuged at 2500*g* at r.t. for 10 min. The resulting cell pellet was washed two times with 1.0 ml of PBS. The cells were resuspended in 2.0 ml of the PBS buffer containing 2.5 mM ONPG, and 200 μl of this suspension were added to a well of 96-well plate to obtain solution with OD_600_ 0.25. A volume of 2.0 μl of DMSO solution without compounds (negative control), DMSO stock solution containing 3 mM of test compounds (final concentration 30 μM, final DMSO concentration is 1%) or 2.0 μl of 1 mM polymyxin B sulfate aqueous solution (final concentration 10 μM, positive control) was added to the mixture. The increase in absorbance at 405 nm triggered by hydrolysis of ONPG inside the cell was measured every 2 minutes for 60 minutes at 37 °C. The values are presented as absorbance after 10, 30 and 60 min of the first measurement with standard deviations obtained in three independent experiments.

To test the possible interactions of silver compounds with β-galactosidase, the cells were lysed in 200 μl of lysis buffer (50 mM Tris·HCl, 2 mM EDTA, 100 mM NaCl, 20 mM sodium β-glycerophosphate, 0.1% Triton X-100) in an ultrasonic bath (240 Hz, 65 W) for 5 min at r.t. The resulting solution was mixed with 1800 μl of PBS containing 2.5 mM ONPG, and 200 μl of this mixture was added to a well of 96-well plate. A volume of 2.0 μl of DMSO solution without compounds (negative control), DMSO stock solution containing 3 mM of test compounds (final concentration 30 μM). The increase in absorbance at 405 nm triggered by hydrolysis of ONPG inside the cell was measured every 10 minutes for 60 minutes at 37 °C. The values are obtained in three independent experiments.

### Statistical analysis

All experiments are performed in triplicate, and data are expressed as mean ± standard deviation. Values were evaluated by two-tailed Student's *t*-test and compared by 95% Tukey's HSD test, using Origin Pro (OriginLab Corp.), n.s. = non-significant, * = *p* < 0.05, ** = *p* < 0.01, *** = *p* < 0.001.

## Data availability

The data supporting this article have been included as part of the manuscript and the ESI.†

## Conflicts of interest

There are no conflicts to declare.

## Supplementary Material

RA-015-D4RA08093A-s001
