# Supplementary material for: Modulation of the mechanism of action of antibacterial silver N-heterocyclic carbene complexes by variation of the halide ligand
Source: RSC Adv. 2025 Jan 20;15(3):1782–91. doi: 10.1039/d4ra08093a (PMC11744769; doi:10.1039/d4ra08093a)
Supplement: RA-015-D4RA08093A-s001 [file RA-015-D4RA08093A-s001.pdf]

Supplementary Information (SI) for RSC Advances.
This journal is © The Royal Society of Chemistry 2025

Supporting Information

Modulation of the mechanism of action of antibacterial silver N-heterocyclic carbene complexes by variation of the halide ligand

Igor V. Esarev, Ingo Ott

Institute of Medicinal and Pharmaceutical Chemistry, Technische Universität Braunschweig, Beethovenstr. 55, 38106 Braunschweig, Germany

Table of contents

Figure S1. The hydrolysis of ONPG in E.coli cell lysate (S2)

Table S1 The optimized temperature program for HRCS-AAS quantification of silver amount in *E.coli* cells (S2)

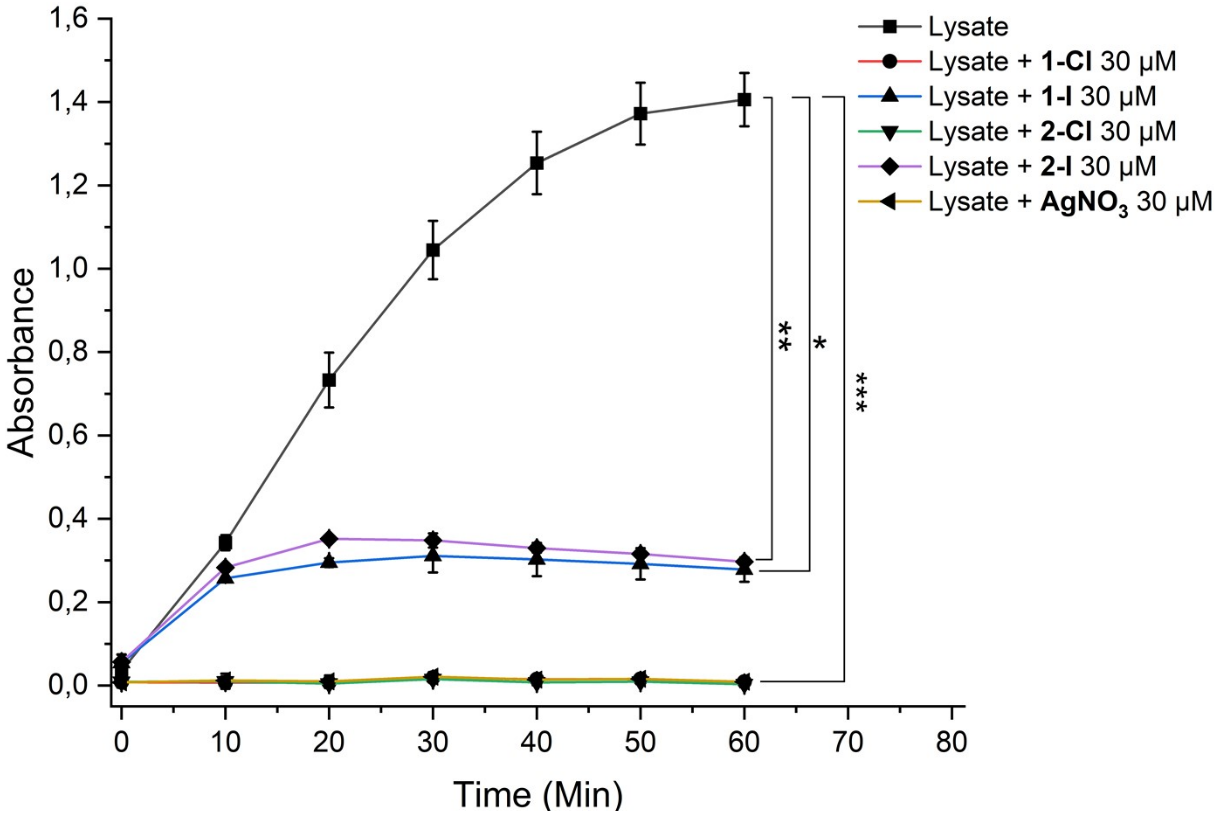

Figure S1. The hydrolysis of ONPG in *E.coli* cell lysate. 200 µl of lysate containing ONPG was mixed with 2 µl of DMSO (Lysate) or 2 µl of 3 mM solutions of chloride (**3a**, **4a**), iodide (**3c**, **4c**) silver NHC complexes or silver nitrate ($AgNO_3$) in DMSO (n = 3). Each sample is compared with cell lysate without treatment by 95% Tukey's HSD test (* = $p < 0.05$, ** = $p < 0.01$, *** = $p < 0.001$)

Table S1. The optimized temperature program for HRCS-AAS quantification of silver amount in *E.coli* cells

Step	Temperature, °C	Heating rate, °C s^{-1}	Hold time, s
Drying	80	6	20
Drying 2	110	5	10
Pyrolysis	350	50	10
Pyrolysis 2	700	300	10
Gas adjustment	700	0	3
Atomization	1600	1500	3
Cleaning	2450	500	4
